# Intersecting risks: A longitudinal analysis of climate exposure, social vulnerability, and mental health in the Texas Gulf Coast

**DOI:** 10.3389/fpubh.2025.1701671

**Published:** 2025-12-10

**Authors:** Lulu Xu, Omolola E. Adepoju, Mary E. Tipton, Hanadi Rifai

**Affiliations:** 1 Humana Integrated Health Systems Sciences Institute, University of Houston, Houston, TX, United States; 2 Tilman J. Fertitta Family College of Medicine, University of Houston, Houston, TX, United States; 3 Hurricane Resilience Research Institute, University of Houston, Houston, TX, United States

**Keywords:** climate health, SVI, mental health, projections and predictions, Texas (TX)

## Abstract

This study investigates the evolving intersection of social vulnerability, climate exposure, and mental health distress in Harris County, Texas, from 2014 to 2022, with projections through 2030. Using census tract-level data and integrating indicators from the CDC Social Vulnerability Index, climate exposure metrics, and mental health outcomes, we developed predictive models to forecast future trends. Results indicate a significant rise in overall social vulnerability—from a score of 8.16 in 2022 to 8.87 by 2030, driven by increasing poverty, extreme heat, flooding, and deteriorating mental health. Mental health distress is projected to escalate from 18.7.0% to 22.7% of the population, underscoring a growing public health burden. The study highlights the compounding risks faced by underserved communities and emphasizes the need for targeted planning, mental health workforce expansion, and adaptive infrastructure strategies. These findings offer critical insights for policymakers and public health officials aiming to mitigate future climate-related health disparities.

## Introduction

In recent years, the frequency and intensity of extreme weather events has increased, causing greater damage to communities, infrastructure, and ecosystems (1). Indexing has emerged as an approach to geospatially characterize aspects of climate change and its impact of the environment. Examples can be found in the Center for Disease Control Social Vulnerability Index (2), the Climate Vulnerability Index (3); and more recently, the Environmental Justice Index (4).

While extreme weather events are equal opportunity events, their impact on underserved communities are disproportionately exacerbated (5), with such communities reporting higher vulnerability indices than neighboring ones. These impacts are products of a combination of structural inequities within the community and unequal responses post weather events. For example, in the Texas Gulf coast, underserved communities tend to lose power first during a hurricane or severe flooding event, and have repair crews work to restore power only after other, more affluent communities, have had their power restored (6, 7). Communities of color are also three times as likely to reside in nature-deprived areas as compared to their white counterparts, they are more susceptible to the deleterious impacts of extreme weather events (6–8).

One example is the historically redlined neighborhoods in California, which experience higher pollution and increased surface temperatures due to fewer green spaces (9). Although resources, such as cooling centers, may be available amidst extreme weather-related hazards, many of these communities are not able to access them because they are not within walking distance and are insufficiently distributed across underserved areas (10, 11). Additionally, longstanding systemic issues both structural and social, including: under-resourced infrastructure like stormwater drainage systems, limited access to healthcare, and fewer economic and social safety nets; all compound these communities’ risk and hinder recovery efforts (12–14).

Notably, the positive association of climate exposure and the risk of developing mental health distress is well-documented (15, 16). Climate-related weather events often result in prolonged disturbances to living conditions in underserved communities, triggering or exacerbating psychological distress for individuals in these areas (17). For example, migrants and refugees experience heightened distress after climate-related events, as these events can intensify the trauma they endured in the past (17). Work by Li et al. (18) mapped social vulnerability indicators to climate change and identified associated health outcomes—such as post-traumatic stress and other mental health disorders—across the literature. Walinski et al. (19) further support these findings, suggesting extreme weather events increase the risk of affective and anxiety disorders, particularly post-traumatic stress disorder, and that chronic exposure to heat increases morbidity and mortality attributed to anxiety disorders.

Recent research suggests that underserved communities often have limited access to resources addressing the mental health distress associated with extreme weather events (17). In rural and low-income areas, there is a limited availability of physicians, and healthcare responses are often delayed (17, 20). Individuals in these areas typically face transportation and linguistic barriers in accessing mental health care (21, 22). Such disparities lead to a lack of culturally tailored responses specific to different underserved populations in the aftermath of climate-related hazards (23).

While prior research has elucidated the relationship between social vulnerability and poor mental health outcomes (15), there is a paucity of studies projecting the mental health burden if current social vulnerability trends continue. Projection studies are useful planning tools to analyze future social vulnerability trends and provide critical insights for policymakers, emergency planners, and public health officials. Using Census and Social Vulnerability Index (SVI) data from 2014 to 2022, this study projects future trends in social vulnerability and the associated mental health burden from 2023 to 2050. Forecasts of this nature are valuable for informing policy decisions related to healthcare planning, resource allocation, and the prioritization of preventive and treatment interventions—particularly for populations expected to experience the greatest increases in social vulnerability.

## Methods

### Data sources

Harris County, which encompasses the metropolitan area of Houston, Texas, is the third largest city in the United States, with over half of its population identifying as Black or Hispanic (24). Located on the coast of the Gulf of Mexico, the area is particularly susceptible to hurricanes and flooding, with multiple major hurricanes causing billions of dollars in damage in the 21st century (25). Since the early 1980s, the area has been subject to over 20 federally declared disasters for severe storms, tropical storms, hurricanes, and major flooding events (26). The number and cost of these events have led the Federal Emergency Management Agency (FEMA) to rank Harris County as having the highest risk of being impacted by hurricanes with the expected annual loss ranked in the 99th percentile and community resilience in the 13th percentile when compared to the rest of the United States (27). In addition to the high amount of weather events within the area, Harris County has a large community of underserved individuals with over 15% of residents living in an economically distressed ZIP code (28). These compounding stressors make Harris County an ideal area for analysis.

Data was obtained from three datasets: (a) SVI, (b) CDC Environmental Public Health Tracking, and (c) Houston State of Health reports. We integrated SVI datasets across five time points—2014, 2016, 2018, 2020, and 2022—focusing on Harris County and key demographic and socioeconomic indicators at the census tract level. Climate exposure was incorporated through three domains: (1) drought, measured by the number of non-consecutive weeks under drought conditions (non-consecutive weeks) from the U.S. Drought Monitor (29); (2) flood risk, two variables defined by the number of days with more than 1 inch and more than 2 inches of rainfall, sourced from the CDC Environmental Public Health Tracking (30); and (3) heat risk, represented by the annual number of days exceeding 95 °F. Mental health outcomes were measured using census tract-level estimates of the percentage of adults reporting poor mental health for more than 14 days in the past month. These estimates were obtained from the *Houston State of Health* (30) reports and aligned with the five SVI and climate data points.

Using these sources, we created a combined dataset at the census tract level for Harris County, integrating SVI scores, climate exposure metrics, and mental health indicators across the five selected years. This longitudinal dataset provided a framework to examine the interactions between social vulnerability, climate stressors, and mental health burden over time.

### Modeling and spatial analysis

Before modeling, we processed the dataset by removing rows with missing values. Missing values for four climate exposure variables (number of days with at least 1 inch of rainfall, number of days with at least 2 inches of rainfall, number of days with temperatures exceeding 95 °F, and the number of weeks classified as moderate drought or worse) were replaced with zeros, assuming no exposure. These three domains were selected because they are the most relevant to Harris County’s geographic and climatic profile. Given the county’s subtropical climate, low elevation, and proximity to the Gulf of Mexico, heat, flooding, and air quality events pose the greatest and most frequent risks to population health. All continuous predictors were standardized to facilitate interpretation of effect sizes and model convergence. We also applied multicollinearity checks (VIF < 10) to remove highly correlated predictors. The final dataset included 4,570 complete observations (2014–2022) with variables across demographics, climate, mental health, and all four SVI themes.

We applied two-way fixed-effects panel regression models (PanelOLS with Driscoll–Kraay robust errors) to examine temporal and cross-sectional associations between overall social vulnerability, mental-health distress, and climate exposures across 2014–2022. Overall social vulnerability was the target outcome in the first model, while the second model employed mental health distress. Thereafter, we plotted regression coefficient plots to show effect sizes with 95% confidence intervals.

To evaluate potential future changes, we applied a bootstrapped linear trend model to illustrate potential changes in overall social vulnerability and mental health distress through 2030. Using overall mean values from 2014 to 2022, we fit simple linear regressions and generated 1,000 bootstrap resamples of model residuals to estimate 95% confidence intervals for projected trends. This descriptive approach provides an uncertainty-aware visualization of temporal patterns, if relationships observed from 2014 to 2022 remain stable through 2030.

Next, to conduct spatial analyses, we linked the tract-level dataset to the 2020 U.S. Census TIGER/Line shapefiles using the FIPS codes for Harris County. Then we averaged variable across 2014–2022 to generate inputs for mapping and spatial autocorrelation tests. We used Global Moran’s I to assess overall spatial autocorrelation in social vulnerability, mental-health distress, and climate exposure variables across Harris County census tracts. Moran’s I quantifies the degree to which similar values cluster in space, ranging from −1 (perfect dispersion) to +1 (perfect clustering), with values near 0 indicating spatial randomness. To identify localized patterns of clustering, we applied Local Moran’s I (LISA) statistics. LISA decomposes the global measure into individual tract-level indices, identifying statistically significant high–high (hot spot) and low–low (cold spot) clusters, as well as high–low and low–high spatial outliers. Overlay analyses were then used to detect overlapping cluster patterns between social vulnerability, mental-health distress, and climate exposures, highlighting where co-occurring risks are spatially concentrated within the county.

## Results

### Modeling of overall social vulnerability

**Figure 1:**
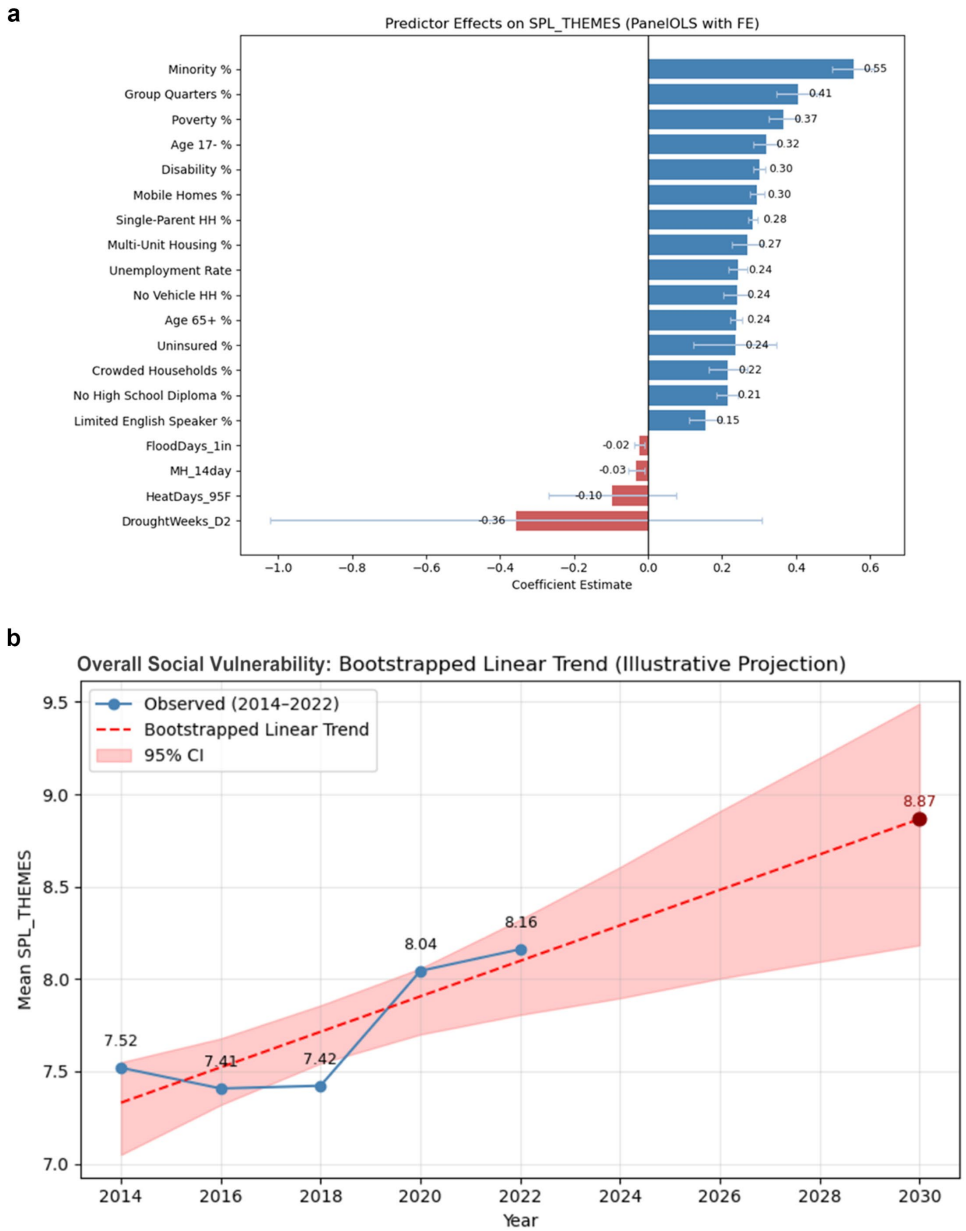
**(a)** Coefficient estimates from two-way fixed-effects panel regression of social vulnerability (SPL_THEMES). **(b)** Observed and bootstrapped linear trend projection of overall social vulnerability (SPL_THEMES), 2014–2030. Observed mean values of overall social vulnerability from 2014 to 2022 are shown in blue. The red dashed line represents the bootstrapped linear trend projection through 2030, with the shaded area indicating 95% confidence intervals based on 1,000 bootstrap resamples.

The result of the two-way fixed-effects panel regression shows a substantial proportion of variation in overall social vulnerability (*R*^2^ = 0.63). Minority proportion showed the strongest positive association with overall social vulnerability (*β* = 0.55), followed by group-quarters share (*β* = 0.41) and poverty rate (*β* = 0.37). Most sociodemographic predictors were positively and significantly associated with higher SVI (*p* < 0.001). Mental health distress was negatively associated with overall vulnerability (*β* = −0.03, *p* = 0.004), although the effect size was small. Among climate exposures, only flood frequency was negatively associated with vulnerability (*p* = 0.002), while heat and drought measures were nonsignificant. In summary, the structural and socioeconomic factors remained the dominant drivers of communit*y*-level vulnerability, mental-health and climate influences playing comparatively minor roles during 2014–2022 (Figure 1a).

The bootstrapped linear trend projection (Figure 1b) indicates a continued increase in overall social vulnerability through 2030. Based on observed means from 2014 to 2022, the model suggests a gradual upward trajectory, with the 2030 overall social vulnerability reaching 8.87 (95% CI: approximately 8.2–9.5). Although the observed values show short-term fluctuations—most notably a sharp rise after 2018—the overall pattern points toward persistent and widening vulnerability across years. The shaded confidence band highlights the uncertainty associated with the limited number of time points, emphasizing that these results represent illustrative trends rather than formal forecasts.

### Modeling of mental health distress

**Figure 2:**
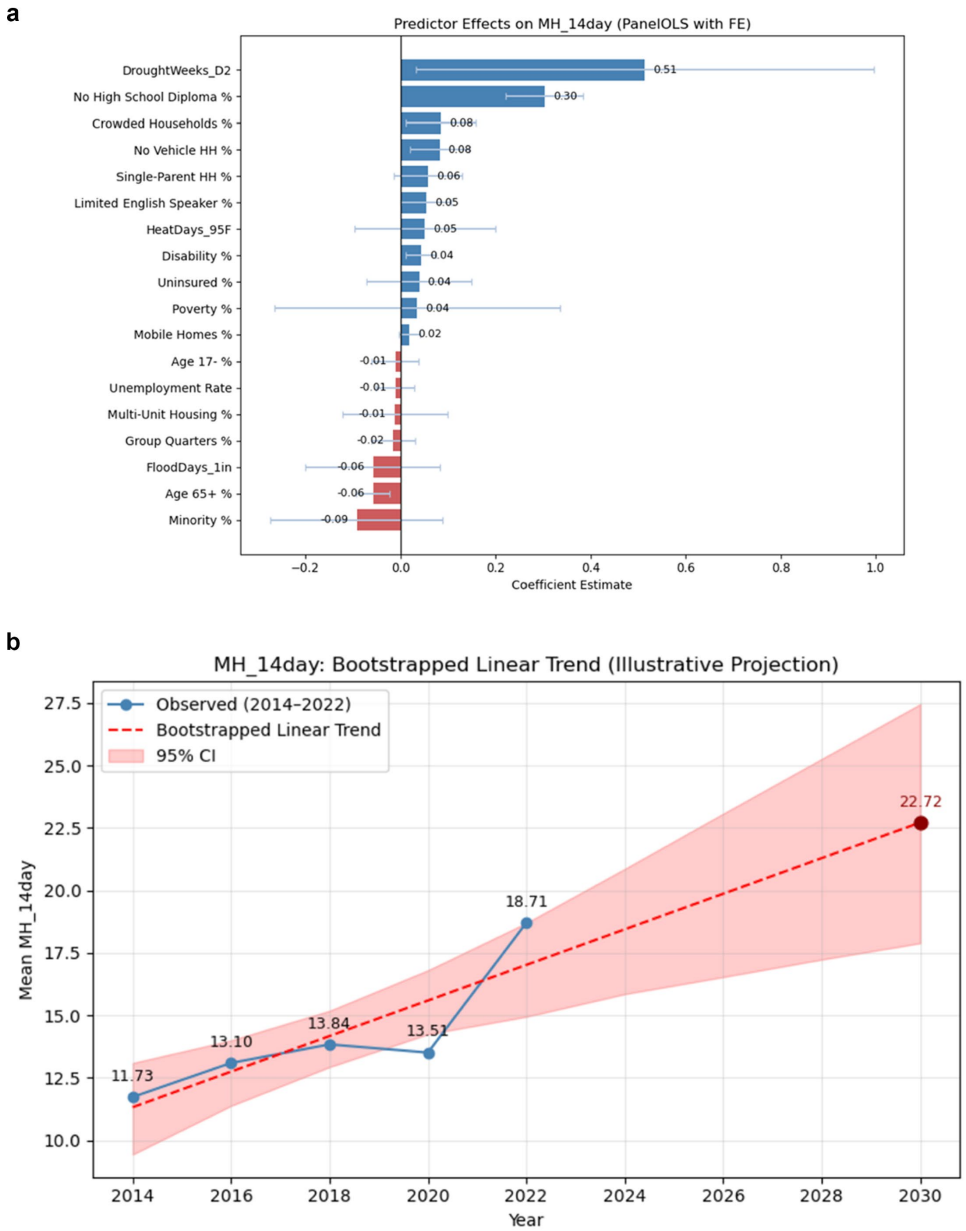
**(a)** Coefficient estimates from two-way fixed-effects panel regression of Mental Health Distress (MH_14plus). **(b)** Observed and bootstrapped linear trend projection of poor mental health (MH_14day), 2014–2030. Observed mean values of Poor Mental Health from 2014 to 2022 are shown in blue. The red dashed line represents the bootstrapped linear trend projection through 2030, with the shaded area indicating 95% confidence intervals based on 1,000 bootstrap resamples.

Results from the two-way fixed-effects panel regression on mental health distress show significant associations between several socioeconomic characteristics and reported distress levels. The overall model explained about 67% of the total variation in mental-health prevalence (*R*^2^ = 0.67). Areas with higher proportions of adults lacking a high-school diploma, crowded housing, households without vehicles, limited-English speakers, and individuals with disabilities reported higher rates of poor mental health (all *p* < 0.05). In contrast, census tracts with a greater proportion of older adults (age 65+) were associated with lower reported mental-health distress. Among climate exposures, only drought duration showed a significant positive relationship with poor-mental-health prevalence; meanwhile, flood and heat days were not significant. Although several predictors reached statistical significance, the relatively wide confidence intervals—particularly for climate variables—suggest variability across tracts and years, indicating that some associations should be interpreted with caution (Figure 2a).

Figure 2b shows a continuing upward trajectory in mental-health distress, with the mean prevalence projected to rise from 18.71% in 2022 to approximately 23% by 2030 (95% CI: about 18%–27%). The widening confidence band reflects growing uncertainty over time, indicating that these projections should be interpreted as illustrative trends rather than precise forecasts.

### Spatial autocorrelation analysis

Global spatial autocorrelation analysis revealed strong and statistically significant clustering across social vulnerability, mental-health distress, flood, heat exposures and drought exposures (*p* < 0.01). This suggests that these variables were not randomly distributed across Harris County. Social vulnerability (Moran’s *I* = 0.702) and poor-mental-health prevalence (*I* = 0.564) showed pronounced spatial dependence, while flood (*I* = 0.880) and heat exposures (*I* = 0.660) exhibited particularly strong spatial concentration. Drought exposure, however, demonstrated weaker but still significant spatial autocorrelation (*I* = 0.312).

**Figure 3:**
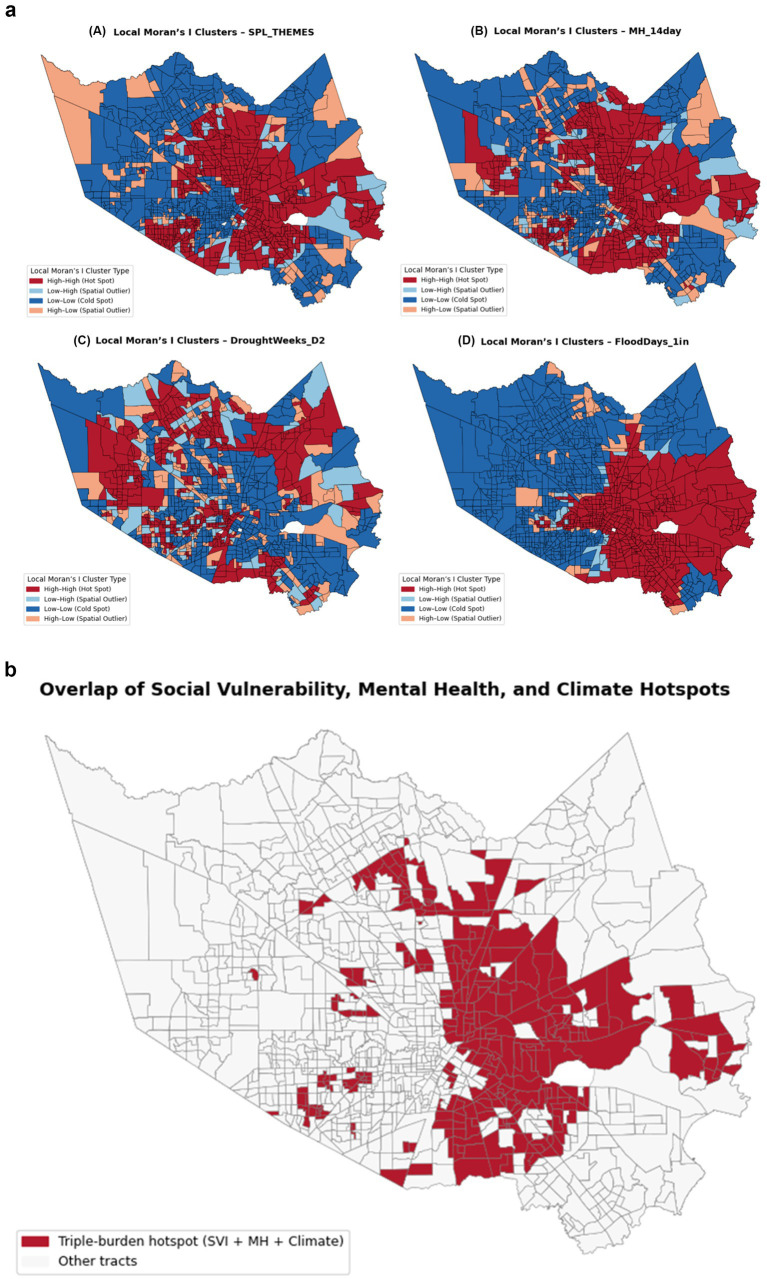
**(a)** Local spatial clusters of social vulnerability, mental health, and climate exposures in Harris County (2014–2022) (panels show Local Moran’s I (LISA) cluster maps for (A, upper left) overall social vulnerability (SPL_THEMES), (B, upper right) poor mental-health prevalence (MH_14day), (C, lower left) drought exposure (DroughtWeeks_D2), and (D, lower right) flood exposure (FloodDays_1in). Red areas denote high–high (hot spot) clusters, blue areas indicate low–low (cold spot) clusters, and light coral or light blue areas represent high–low and low–high spatial outliers, respectively). **(b)** Overlapping Hotspots of Social Vulnerability, Mental Health, and Climate Exposures in Harris County.

Local Moran’s I (LISA) cluster maps results revealed statistically significant spatial clustering patterns for (A) overall social vulnerability, (B) poor mental-health prevalence, (C) drought exposure, and (D) flood exposure across Harris County census tracts (Figure 3a). Across all four maps, SVI and poor-mental-health hot spots were over-represented in the inner-city and east/southeast regions, while drought exposure hot spots were primarily located in the western and northern parts of the county. Flood-related hot spots were primarily concentrated in the southern and eastern portions of the county.

The overlap map (Figure 3b) displays census tracts simultaneously identified as high–high (hot spot) clusters for social vulnerability, poor mental-health prevalence, and at least one climate stressor (flood or drought exposure). Red areas represent triple-burden hotspots, where SVI, mental-health distress, and climate risk co-occur, while gray tracts indicate areas without overlap. A total of 986 tracts, covering a substantial portion of Harris County, were identified as triple-burden areas, indicating substantial spatial convergence of multiple vulnerabilities. These hotspots are concentrated in central, eastern, and southern Harris County.

## Discussion

In this analysis of social vulnerability, climate exposure, and mental health distress in Harris County, our findings suggest that overall social vulnerability will increase significantly by 2030, under conservative climate and health scenarios. This trend is concordant with work analyzing vulnerability indices across metropolitan Texas, which found an increase in vulnerable neighborhoods from a previous period of 2000 to 2018 (31). Additionally, analysis of Climate Vulnerability Index (CVI), a nationwide index that combines climate change impacts with traditional indicators of vulnerability, across the U.S. particularly notes Harris County as a case example of an area with distinct geographic climate vulnerability disparities (3). Given that both social vulnerability and climate change are contributors to factors increasing adverse health conditions (32, 33) and worsened mental health outcomes (34, 35), the continued proliferation of social vulnerability, particularly among existing vulnerable populations in Harris County raises high concern. As areas continue to experience changes from increasing development and diversity (36) in the rise of climate change, identifying the driving forces of social vulnerability is essential in creating hazard assessment and emergency preparedness (36) for natural disasters.

Mental health distress also showed a strong upward trend in 2030, with the proportion of people reporting greater than 14 days of mental distress reaching 23% of the population. This finding has significant implications for the mental healthcare workforce, particularly in the Greater Houston area, which is already experiencing a mental health care shortage (37). One in 4 Harris County residents reporting greater than 14 days of mental health distress in a month highlights the growing demand for climate-related and preventative mental health resources for communities across the region. Others have suggested community Mental Health First Aid (MHFA) programs as a way to bridge the current and projected workforce gaps (38). While there are multiple programs and interventions that aim to address mental health (39, 40), MHFA in particular focuses on equipping individuals already embedded within a community with the tools and knowledge to be a conduit to accessing behavioral health care (41). While this program is not a complete answer to the mental health care shortage, it can be a first step in capacity building. Implementation of this program during non-event ‘blue sky’ periods would allow for trainees to spread the knowledge gained in the program to others within their community, helping to increase resiliency when coping with the stressors that come with weather events. Adult MHFA training focuses on helping participants—many of whom are parents, caregivers, teachers, clergy, and nonprofit staff—identify early warning signs of mental health conditions in adults and guide individuals toward appropriate support (42). Youth MHFA training specifically prepares adults to recognize and respond to mental health challenges in children and adolescents, with an emphasis on developmentally appropriate interventions and reducing stigma around youth mental health (43). A critical component of this training includes teaching participants how to refer individuals in distress to a behavioral health medical home. This program, which consists of eight hours of high-yield topics, is designed for flexibility in administering training sessions, with options for delivery entirely in person, entirely virtual, or a blend of both (44). This flexibility allows for training location, timing, and length to be tailored to the needs of the community.

Key drivers of increased vulnerability included socioeconomic factors, more frequent extreme heat and flooding, and deteriorating mental health. Work mapping social vulnerability indices amid climate change agrees with these findings, linking lower socioeconomic level and pre-existing mental or cognitive health conditions with decreased resilience to health impacts from climate change (18, 45). Moreover, there is linkage between natural disaster events and vulnerability-related factors, including population growth and distribution and social diversity (46). These findings are especially concerning under Harris County’s rising vulnerability, considering that the extreme heat and flooding events are only predicted to increase in frequency (47). The widespread “hot-spotting” overlap underscores the compounded nature of social, health, and environmental risks in these communities and highlights the need for integrated resilience and public-health interventions targeting the most burdened areas.

This study is not without limitations. The analysis is at the census tract level, which, while useful for identifying community-wide trends, may obscure important individual-level variations. Aggregating data at this geographic scale can mask disparities within tracts and lead to ecological fallacies—where assumptions about individuals are drawn from group-level data. As a result, our projections may be subject to bias and may not fully capture the nuanced experiences of individuals, particularly those in highly heterogeneous or rapidly changing communities. Additionally, health effects of flooding may manifest weeks or months after exposure, and influence observed associations; however, our multi-year analytic approach helps account for these delayed impacts and reduce potential bias. Considerations for future work include the utilization and comparison of alternative vulnerability subindices, such as the climate vulnerability index (48), or subindices related to specific climate concerns such as community resilience estimates for heat (49). In addition, the results of this study should be generalized with caution. Generalizing these results outside of coastal urban areas may lead to misinterpretation and ineffective policy decisions.

Despite these limitations, there are many strengths in the study. Multiple links were established in this novel study analyzing the intersections between social vulnerability, climate exposure, and mental health distress in Harris County. Given the large, vulnerable populations and rising successive extreme weather-related disaster events (47) the region will continue to face, it is key to analyze indicators of vulnerability and establish community screening, assessment, and interventions to facilitate climate adaptation and health protection.

## Conclusion

This study demonstrates that social vulnerability and mental health distress in Harris County are both influenced by a combination of demographic, environmental, and climate-related factors. Using a robust, integrated dataset spanning 2014 to 2022, we applied linear regression models and scenario-based forecasting to project future trends under conservative assumptions. Results suggest that overall social vulnerability (SPL_THEMES) is likely to increase significantly by 2030, primarily due to rising poverty, climate extremes, and mental health burdens. Mental health distress is also projected to rise, underscoring an increasing need to address the pre-existing gaps in mental healthcare. These projections are an urgent call for policymakers and health officials at all levels to act now to ameliorate the impacts that climate-related disparities have on the most vulnerable communities.

## Data Availability

Publicly available datasets were analyzed in this study. This data can be found at: https://www.atsdr.cdc.gov/place-health/php/svi/svi-data-documentation-download.html, https://ephtracking.cdc.gov/Applications/heatTracker/, and https://www.houstonstateofhealth.com/tiles/index/display?id=167554482011231603.
